# AnomNet: A Dual-Stage Centroid Optimization Framework for Unsupervised Anomaly Detection

**DOI:** 10.3390/jimaging11090301

**Published:** 2025-09-03

**Authors:** Yuan Gao, Yu Wang, Xiaoguang Tu, Jiaqing Shen

**Affiliations:** 1 College of Aviation Electronic and Electrical Engineering, Civil Aviation Flight University of China, Chengdu 641450, China; gaoyuan@cafuc.edu.cn (Y.G.); 18170271693@163.com (Y.W.); 13881017769@163.com (J.S.); 2 Sichuan Province Engineering Technology Research Center of General Aircraft Maintenance, Civil Aviation Flight University of China, Guanghan 618307, China

**Keywords:** anomaly detection, domain adaptation, contrastive learning, industrial inspection, unsupervised learning

## Abstract

Anomaly detection plays a vital role in ensuring product quality and operational safety across various industrial applications, from manufacturing to infrastructure monitoring. However, current methods often struggle with challenges such as limited generalization to complex multimodal anomalies, poor adaptation to domain-specific patterns, and reduced feature discriminability due to domain gaps between pre-trained models and industrial data. To address these issues, we propose AnomNet, a novel deep anomaly detection framework that integrates a lightweight feature adapter module to bridge domain discrepancies and enhance multi-scale feature discriminability from pre-trained backbones. AnomNet is trained using a dual-stage centroid learning strategy: the first stage employs separation and entropy regularization losses to stabilize and optimize the centroid representation of normal samples; the second stage introduces a centroid-based contrastive learning mechanism to refine decision boundaries by adaptively managing inter- and intra-class feature relationships. The experimental results on the MVTec AD dataset demonstrate the superior performance of AnomNet, achieving a 99.5% image-level AUROC and 98.3% pixel-level AUROC, underscoring its effectiveness and robustness for anomaly detection and localization in industrial environments.

## 1. Introduction

In industrial manufacturing, the quality and reliability of finished products are heavily influenced by technological processes, labor conditions, and other external factors. Anomaly detection plays a pivotal role in ensuring quality control and operational safety [1,2], with extensive applications across visual inspection, defect identification, and real-time monitoring systems. However, anomalies in real-world scenarios are often rare, diverse, and unpredictable, making it impractical to enumerate all potential types during training. As a result, constructing comprehensive labeled datasets that cover all possible abnormal cases is infeasible. This motivates the adoption of learning-from-normal paradigms, where models are trained exclusively on normal samples and anomalies are identified as deviations from learned patterns.

Several learning paradigms have been proposed for anomaly detection, including supervised, semisupervised, and unsupervised methods. Among these, semisupervised learning [3], which combines a small amount of labeled anomaly data with a large volume of unlabeled data, has demonstrated strong detection performance. However, in many industrial scenarios, acquiring labeled anomaly data is often prohibitively expensive and impractical. Therefore, unsupervised learning, due to its independence from such labels, has become the mainstream approach in this domain [4]. These methods rely on raw, unlabeled inputs and often leverage reconstruction-based objectives, adversarial learning, or selfsupervised feature learning to model the distribution of normal data. During inference, deviations from the learned normal patterns are interpreted as anomalies, typically modeled as out-of-distribution (OOD) features [5]. Existing unsupervised anomaly detection techniques can be broadly categorized into three groups: reconstruction-based, generation-based, and embedding-based approaches.

Reconstruction-based methods were among the earliest approaches to gain widespread attention and application in the development of unsupervised anomaly detection.These methods attempt to reconstruct normal inputs and utilize the reconstruction error to identify anomalies [6,7,8]. When an anomalous input is fed into the model, it tends to “hallucinate” normal regions, leading to residuals that highlight defective areas. Traditional reconstruction models include autoencoders (AE) [9], which consist of an encoder that maps input to a low-dimensional latent space and a decoder that reconstructs the original data. Their broad applications in machine learning have been extensively surveyed [10]. Youkachen et al. [11] proposed a convolutional autoencoder-based approach for industrial image reconstruction, successfully segmenting surface defects on hot-rolled strips by highlighting differences between input and reconstructed images. Mei et al. [12] designed a Multi-Scale Convolutional Denoising Autoencoder (MSCDAE), integrating multi-pyramid layers and multimodal reconstruction results to improve detection accuracy across various material types. However, these methods often struggle with fine-grained structures or high-frequency textures, where reconstruction quality deteriorates, thus impairing anomaly localization performance.

To overcome the limitations of autoencoders in reconstruction performance, generation-based methods were introduced to anomaly detection tasks. These methods employ generative models to learn the underlying distribution of normal data and generate samples accordingly [13,14,15]. Anomalies are detected by evaluating discrepancies between generated and observed data in either pixel or latent space. Generative Adversarial Networks (GANs) [16], consisting of a generator and discriminator, have been particularly effective in this domain. The pioneering AnoGAN [13] introduced unsupervised anomaly detection using deep convolutional GANs, where the generator learns a manifold of normal data distribution and anomalies are identified through a novel scoring scheme based on reconstruction and discrimination errors. Building upon this foundation, GANomaly [14] proposed a semisupervised approach that employs an encoder–decoder–encoder architecture to jointly learn image generation and latent space inference, achieving improved detection performance through adversarial training. Liu et al. [15] combined GAN with a one-class classifier for steel surface anomaly detection. Additionally, normalizing flow-based methods such as CFLOW [17], FastFlow [18], and CS-Flow [19] transform complex feature distributions of normal samples into Gaussian distributions, enabling efficient anomaly detection. While these methods provide improvements over basic reconstruction techniques, they still face challenges in capturing fine-grained discriminative information in the feature space.

Diffusion models have recently demonstrated remarkable generative and reconstruction capabilities [20], and their applications have been extended to unsupervised anomaly detection. Early studies primarily leveraged the reconstruction ability of diffusion models on normal samples, where the reconstruction error served as a criterion to distinguish anomalies from normal data [21]. Subsequent research introduced latent-space diffusion methods such as DiffAD, which leverages a latent diffusion model equipped with noisy-condition embedding and interpolated channels to prevent direct reconstruction of anomalies and enhance both anomaly detection and localization performance [22]. More recent advances explored continual diffusion formulations, such as the One-for-More model [23], which dynamically adapts to novel categories while mitigating issues of faithfulness hallucination and catastrophic forgetting, thus enhancing the adaptability and generalization of unsupervised anomaly detection in open-world environments. Despite their generative power, these models are often limited by high computational costs and the inherent risk that their strong reconstruction ability can paradoxically undermine anomaly detection.

While reconstruction and generation-based methods have achieved notable success, a core challenge remains their limited discriminative power in the feature space. Embedding-based methods have recently demonstrated superior detection performance and generalization capabilities [19,24,25,26]. These approaches utilize pre-trained convolutional neural networks (CNNs), such as those trained on ImageNet, to extract semantic features from input images. Normal feature distributions are then embedded and stored, and anomaly scores are computed based on the distance between test samples and these stored embeddings. This paradigm of storing representations of normal data conceptually mirrors memory networks used in other domains, such as knowledge tracing, where robust representations of past states are crucial for future predictions [27]. PaDiM [24] models feature distributions at each spatial location with multivariate Gaussian distributions and computes anomaly scores using the Mahalanobis distance. Cohen et al. [28] proposed SPADE, which applies a k-nearest neighbor (KNN) search in feature space for anomaly detection. Building upon this, Roth et al. [25] developed PatchCore, incorporating a memory bank with coreset subsampling to significantly reduce storage requirements while maintaining competitive performance.

Within the embedding-based paradigm, another important branch leverages knowledge distillation frameworks for anomaly detection. The foundational “uninformed student” model, introduced by Bergmann et al. [29], trains a student network to replicate the output of a pre-trained teacher on normal samples, with anomalies localized through discrepancies between their outputs. This approach has been refined in subsequent work. For example, a multi-resolution strategy was developed to better capture semantic deviations by leveraging features from multiple intermediate layers [30], while localization precision was enhanced through a feature pyramid matching mechanism [31]. More recently, the MemKD [32] framework was employed to address the “normality forgetting” problem, thereby enhancing the student model’s memory capability for normal patterns.

Despite their effectiveness, embedding-based methods face a critical limitation: the significant domain shift between natural image datasets (e.g., ImageNet) and industrial scenarios leads to domain mismatch, undermining feature discriminability and overall performance. This challenge motivates the need for more sophisticated approaches that can better adapt pre-trained features to industrial domains while maintaining the advantages of deep feature representations.

To address the limitations of existing methods, we introduce **AnomNet**, a novel deep learning framework for unsupervised anomaly detection and localization in industrial settings. AnomNet integrates the strengths of embedding-based learning and introduces two key innovations. First, a feature adapter module is proposed to mitigate domain bias between pre-trained backbones and industrial data, enhancing the discriminative capability of extracted features. Second, we propose a dual-stage centroid optimization (DSCO) training strategy. In the first stage, centroids are optimized using separation and entropy regularization losses to model the complex, multimodal distribution of normal features robustly. Once the centroids are well-formed, the model enters a second stage, where we introduce a centroid-based contrastive learning (CCL) mechanism. This stage refines decision boundaries by promoting intra-class compactness and inter-class separation, thereby improving the model’s ability to distinguish between normal and abnormal patterns. Our main contributions are summarized as follows:

- We propose a lightweight feature adapter module to alleviate domain bias between pre-trained backbones and industrial images, enhancing multi-scale feature discriminability without increasing model complexity.
- We design a dual-stage centroid optimization (DSCO) strategy that first stabilizes centroid representations of normal features via separation and entropy regularization, and then refines decision boundaries using centroid-guided contrastive learning to improve anomaly discrimination.
- We achieve state-of-the-art performance on MVTec AD and VisA benchmarks at both image and pixel levels, demonstrating the effectiveness of AnomNet in the task of unsupervised anomaly detection.

## 2. Methods

**Figure 1:**
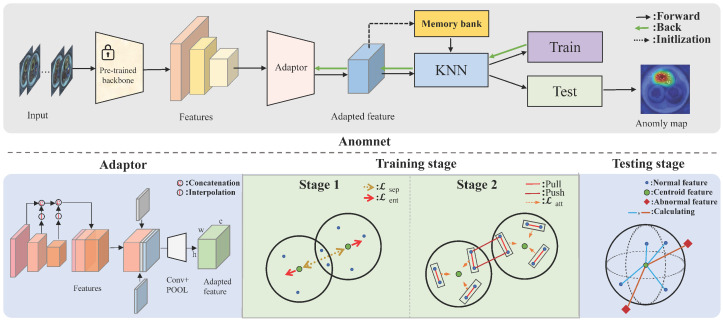
Overview of the AnomNet framework. The input image is first processed by a pre-trained convolutional backbone to extract multi-scale feature representations. These features are then passed through a lightweight feature adapter to mitigate domain discrepancies. The training pipeline is divided into two stages: the first stage focuses on optimizing feature centroids using separation loss and entropy-based regularization; the second stage refines decision boundaries via centroid-guided contrastive learning. During inference, anomaly scores are computed by measuring the distance between extracted features and learned centroids, enabling the generation of pixel-level heatmaps for accurate anomaly localization.

In this section, we present the proposed anomaly detection framework, AnomNet, which integrates dual-stage centroid optimization with contrastive learning [33,34] to substantially improve the discrimination and localization of anomalies. As depicted in Figure 1, the framework consists of three primary components: feature extraction and adaptation, dual-stage centroid learning, and anomaly detection with localization. The dual-stage centroid learning procedure begins by optimizing the positions of centroids to accurately capture the distribution of normal samples. Subsequently, it refines the decision boundaries through contrastive learning, thereby enhancing the model’s ability to distinguish anomalies. This process is supported by a feature extraction and adaptation module designed to reduce domain shift, and concludes with a robust anomaly scoring mechanism that enables precise detection and localization.

### 2.1. Feature Extraction and Adaptation

A major challenge in industrial anomaly detection lies in the substantial domain discrepancy between industrial imagery and the source domain (e.g., ImageNet) on which most pre-trained models are based. To address this issue, we propose a lightweight feature adaptation module designed to mitigate domain shift and facilitate the extraction of more discriminative feature representations.

In our approach, we adopt a deep convolutional neural network pre-trained on ImageNet as the backbone feature extractor. As depicted in the upper portion of the figure, we extract feature maps from multiple intermediate layers of the backbone to capture visual information at various levels of scale and abstraction. These multi-level features encompass both low-level visual cues (e.g., edges and textures) and high-level semantic representations, which are critical for modeling the normal patterns characteristic of industrial products.

To bridge the domain gap between the pre-trained feature representations and those pertinent to the industrial domain, we introduce a feature adaptation module based on coordinate convolution [35] (CoordConv). As illustrated in the lower-left portion of the figure, this adapter module enhances the model’s sensitivity to structural characteristics specific to industrial images by integrating multi-scale features and embedding spatial coordinate information. The key idea behind coordinate convolution is to augment feature maps with explicit spatial coordinate channels, which enhances the network’s ability to capture position-sensitive patterns. This capability is crucial for accurate anomaly localization.

To provide theoretical justification, our adapter’s design is grounded in domain adaptation theory [36]. The theory provides an upper bound for the target domain error $\epsilon_{T}$ as follows:

(1) $$\epsilon_{T}\left(h\right)\leq \epsilon_{S}\left(h\right)+d_{H\Delta H}\left(D_{S},D_{T}\right)+\lambda_{0}$$

where $\epsilon_{S}$ is the source error, $d_{H\Delta H}$ measures the divergence between the source and target feature distributions, and $\lambda_{0}$ is a constant. Our primary goal is to minimize the divergence term $d_{H\Delta H}$. The CoordConv mechanism achieves this by augmenting the original features f with domain-invariant coordinate channels c, creating an enhanced feature $f^{\prime }=\left[f,c\right]$. Since the coordinate distribution P(c) is identical across domains, it serves as a stable anchor, allowing the adapter to learn a mapping that aligns features based on spatial context, thereby effectively reducing the overall distribution gap. This approach ensures robustness across different pre-trained backbones; as long as the backbone provides a rich feature hierarchy, our adapter can effectively reduce the domain divergence, thus implicitly bounding the adaptation loss without requiring a strict, backbone-specific derivation.

For multi-scale feature fusion, we employ adaptive pooling operations to normalize the spatial dimensions of features extracted at different scales. These unified feature maps are subsequently concatenated along the channel dimension, as illustrated in the ConvPOOL module, yielding an adapted feature representation of size c×h×w:

(2) $$F_{\mathrm{adapted}}=\mathrm{Concat}\left(P_{1}\left(F_{1}\right),P_{2}\left(F_{2}\right),\ldots ,P_{n}\left(F_{n}\right)\right)$$

where $F_{i}$ represents the output of the *i*-th feature layer, $P_{i}$ denotes the adaptive pooling operation applied to that layer, and Concat indicates the concatenation operation along the channel dimension. This design enables the feature adapter to effectively capture multi-scale contextual information while simultaneously enhancing the model’s sensitivity to spatial structures through the explicit incorporation of coordinate information. By integrating both scale-aware and position-aware features, the proposed adapter facilitates the generation of more robust and discriminative representations for industrial anomaly detection.

### 2.2. Dual-Stage Centroid Learning

Traditional approaches such as Deep SVDD [37] model the distribution of normal samples using a single centroid and a corresponding hypersphere. While effective in simple scenarios, this overly simplistic representation exhibits substantial limitations when applied to industrial data characterized by complex textures and multimodal surface patterns. To overcome these challenges, we propose a dual-stage centroid learning framework that substantially improves the model’s ability to capture the intricate distribution of normal samples. The proposed method consists of two successive stages: a centroid optimization stage, which refines the representation of normal features, and a contrastive learning stage, which further enhances feature separability by leveraging discriminative representations in a comparative learning setting.

**Centroid Optimization Stage**: In the first stage, the focus is on optimizing the positions and distribution of centroids to effectively capture the multimodal nature of normal sample distributions in the feature space. The centroids are initially initialized using the global mean of the training sample features. To prevent centroid collapse (the convergence of multiple centroids to a single location), we incorporate two essential regularization strategies: separation loss, which encourages diversity among centroids, and entropy regularization loss, which promotes a balanced and informative distribution of feature assignments across centroids.

The separation loss is designed to maintain adequate distances between centroids, thereby preventing collapse. It is formally defined as:

(3) $$L_{\mathrm{sep}}=\frac{1}{|C_{K}|}\sum_{\left(k,l\right)\in C_{K}}\max\left(0,\theta -d\left(C_{k},C_{l}\right)\right)$$

where *K* is the total number of centroids, $d(C_{k},C_{l})$ represents the distance between the *k*-th and *l*-th centroid pair, and θ is the penalty threshold. When the distance between centroids is less than θ, this loss term imposes a penalty to encourage greater separation between them. Entropy regularization promotes centroid diversity by maximizing the variance among centroids and is defined as follows:

(4) $$L_{\mathrm{ent}}=-H\left(\left\{C_{1},C_{2},\ldots ,C_{k}\right\}\right)$$

where H(·) denotes the entropy of the centroid set, which is computed based on Gaussian entropy. Additionally, we introduce an attraction loss to encourage normal sample features to cluster closely around their nearest centroid:

(5) $$L_{\mathrm{att}}=\frac{1}{N}\sum_{i=1}^{N}\min_{k}{∥f_{i}-C_{k}∥}_{2}^{2}$$

where *N* denotes the batch size, $f_{i}$ represents the feature of the *i*-th sample, and the minimization is performed over all centroids $C_{k}$ in the set of K centroids C.

Through the combined effect of these loss functions, the first-stage training effectively distributes the centroids across distinct regions of the feature space, thereby providing a more accurate representation of the complex distribution of normal samples.

**Contrastive Learning Stage**: Following centroid optimization, we advance to the second stage, which utilizes a centroid-based contrastive learning (CCL) mechanism to further refine decision boundaries and strengthen the model’s discriminative capabilities. In this stage, the centroid positions are fixed and serve as anchors to guide the feature learning process. To enhance reproducibility, the core logic of this stage is summarized in Algorithm 1.

**Algorithm 1** Pseudocode for Stage 2 CCL Optimization**Require:** A batch of features F, fixed centroids C, margin *m*.
  1:// Step 1: Assign pseudo-labels  2:**for all** feature $f_{i}\in F\;$**do**  3:      Assign pseudo-label $y_{i}\leftarrow \arg\min_{k}{∥f_{i}-C_{k}∥}_{2}$.  4:**end for**  5:// Step 2: Construct pairs and compute loss  6:Normalize features F to get $F_{norm}$.  7:Compute similarity matrix $S\leftarrow F_{norm}\cdot F_{norm}^{⊤}$.  8:Construct positive pair set $P\leftarrow \{\left(i,j\right)|y_{i}=y_{j},i<j\}$.  9:Construct negative pair set $N\leftarrow \{\left(i,j\right)|y_{i}\neq y_{j},i<j\}$.10:$L_{pos}\leftarrow \frac{1}{\left|P\right|}\sum_{(i,j)\in P}\left(1-S_{ij}\right)$.11:$L_{neg}\leftarrow \frac{1}{\left|N\right|}\sum_{(i,j)\in N}\max\left(0,S_{ij}-m\right)$.12:$L_{con}\leftarrow L_{pos}+L_{neg}$.13:// Step 3: Update model14:Update feature extractor via gradient descent on $L_{con}$.


Our centroid-guided contrastive learning operates in three steps: First, it performs pseudo-label assignment by associating each feature point with its closest centroid; second, it constructs positive pairs from features sharing the same centroid label and negative pairs from those with different labels; finally, it optimizes the feature space via contrastive learning, which encourages intra-class compactness and inter-class separability.

Specifically, the process begins by assigning a pseudo-label to each feature vector based on its nearest centroid, as formulated in Equation (6):

(6) $$y_{i}=\arg\min_{k}{∥f_{i}-C_{k}∥}_{2}$$

where $y_{i}$ denotes the pseudo-label of feature $f_{i}$, and $C_{k}$ is the *k*-th centroid from the set of K fixed centroids C. Based on these pseudo-labels, we construct positive and negative sample pairs: when two feature vectors are assigned to the same centroid ($y_{i}=y_{j}$), they form a positive pair; otherwise ($y_{i}\neq y_{j}$), they form a negative pair. For instance, two feature vectors from a ‘smooth metal’ surface would be assigned the same pseudo-label and form a positive pair to be pulled together, while a feature from a ‘regular texture’ surface would form a negative pair with them and be pushed away.

After applying normalization to all feature vectors, the similarity between feature pairs is computed using cosine similarity:

(7) $$sim\left(f_{i},f_{j}\right)=f_{i}^{⊤}f_{j}$$

For positive pairs, we define the loss to enhance intra-class similarity:

(8) $$L_{pos}=\frac{1}{\left|P\right|}\sum_{(i,j)\in P}\left(1-sim\left(f_{i},f_{j}\right)\right)$$

where *P* denotes the set of positive pairs.

For negative pairs, we introduce a margin-based hinge loss to suppress high similarity between features assigned to different centroids:

(9) $$L_{neg}=\frac{1}{\left|N\right|}\sum_{(i,j)\in N}\max\left(0,sim\left(f_{i},f_{j}\right)-m\right)$$

where *N* denotes the set of negative pairs, and *m* is the predefined margin threshold. The overall contrastive learning loss is defined as:

(10) $$L_{con}=L_{pos}+L_{neg}$$

Through this centroid-based contrastive learning mechanism, the model can learn more discriminative feature representations while more effectively distinguishing between normal and anomalous patterns. To formally ground the stability of this stage, we can frame the optimization through the lens of Lyapunov stability. Let the state of our system be the set of feature embeddings generated by the feature extractor. Our objective is to show that this state converges to a stable configuration. We define the contrastive loss, $L_{\mathrm{con}}$, as our Lyapunov function candidate, V(·), which is non-negative by definition. The fixed centroids act as stable attractors in the feature space. The optimization process, driven by gradient descent, is designed to monotonically decrease the value of $L_{\mathrm{con}}$ at each step (ΔV≤0). Since the loss is bounded below by zero and is non-increasing, it is guaranteed to converge. As the optimization minimizes $L_{\mathrm{con}}$, the feature embeddings are driven towards a stable equilibrium where they are compactly clustered around their respective centroid anchors, ensuring the convergence and stability of the learned feature space. After the second stage of training, the model will form a decision boundary composed of multiple hyperspheres centered at the optimized centroids, which can more precisely delineate the distribution boundary of normal samples.

The total loss function for our dual-stage centroid learning framework can be summarized as:

(11) $$L_{\mathrm{total}}=\left\{\begin{array}{cc} \alpha L_{\mathrm{att}}+\beta L_{\mathrm{sep}}+\gamma L_{\mathrm{ent}}, & \mathrm{Stage}\;1\\ \delta L_{\mathrm{att}}+\lambda L_{\mathrm{con}}, & \mathrm{Stage}\;2 \end{array}\right.$$

where α, β, γ, δ, and λ are weighting coefficients that balance the contributions of the various loss terms in each stage. In the first stage, emphasis is placed on centroid separation and entropy regularization to establish a well-distributed and robust initial configuration of centroids. In the second stage, the focus shifts to feature refinement via contrastive learning, while maintaining strong attraction to the fixed centroids.

Throughout the dual-stage training process, the first stage is dedicated to optimizing centroid positions, thereby forming a preliminary and representative modeling of the normal sample distribution. The second stage further sharpens decision boundaries through contrastive learning, promoting tighter intra-class clustering and greater inter-class separation.

### 2.3. Anomaly Detection and Localization

Upon completion of training via our dual-stage centroid learning framework, the model is capable of accurately detecting and localizing anomalies in test images. During inference, input images are processed through the same feature extraction and adaptation pipeline used in training to generate feature representations, which are then compared against the learned centroids.

For image-level anomaly detection, we calculate the minimum distance between each feature and its closest centroid as follows:

(12) $$d_{\min}\left(f\right)=\min_{k}{∥f-C_{k}∥}_{2}$$

where *f* is a feature vector at a given spatial location, and the minimization is performed over all learned centroids $C_{k}$. This minimum distance serves as the anomaly score for that specific feature point, reflecting its deviation from the normal distribution centers. Features closer to their nearest centroids are more likely to represent normal patterns, while those farther away indicate potential anomalies.

To generate a pixel-level anomaly heatmap, the anomaly score is computed for each feature vector, resulting in a score map with the same spatial dimensions as the feature map. This score map is then upsampled to match the input image dimensions via bilinear interpolation. To suppress local fluctuations and enhance spatial consistency, Gaussian smoothing is applied, followed by min-max normalization to scale the scores within the [0, 1] range. This multi-step processing pipeline ensures precise localization of anomalous regions while effectively reducing noise interference. The normalized heatmap serves as the definitive anomaly distribution map for both evaluation and visualization.

For image-level anomaly detection, global pooling is performed on the heatmap to produce a single overall anomaly score. The image is classified as anomalous if this score exceeds a predefined threshold, allowing for sensitivity adjustments tailored to specific application requirements. For pixel-level defect localization, thresholding is applied to the normalized heatmap to generate a binary segmentation mask that delineates anomalous regions. This dual-output framework enables our method to simultaneously address classification and segmentation tasks, providing valuable insights for downstream applications such as defect categorization and remediation.

## 3. Experiments

### 3.1. Experimental Setup

In this section, we first validate the effectiveness of our proposed dual-stage centroid learning framework on challenging industrial anomaly detection tasks. We then assess the detection performance by comparing our method against several state-of-the-art approaches across diverse categories and domains. For evaluation, we primarily use the area under the receiver operating characteristic curve (AUROC) at both the image and pixel levels, denoted as I-AUROC and P-AUROC, respectively.

(1) Datasets: We conducted extensive evaluations on two challenging datasets to evaluate the effectiveness of our method.

MVTec-AD [38] consists of 5354 images across 15 categories for industrial anomaly detection tasks. It includes 10 object categories and 5 texture categories. There are a total of 3629 normal images serving as the training set, while the test set contains 1725 images (467 normal and 1258 anomalous). The image sizes range from 700 × 700 to 1024 × 1024 pixels.

VisA [39] contains a total of 10,821 high-resolution color images, with 9621 being normal and 1200 being anomalous. It covers 12 objects across three domains, namely, complex structure, multiple instances, and single instances. The dataset is twice the size of MVTec. The anomalous images present a wide range of flaws, including surface defects like scratches, dents, color spots, and cracks, as well as structural defects such as misplacement or missing parts. Each defect type has 5–20 images, and an image might have multiple defects. All images were captured by a 4000 × 6000 high-resolution RGB sensor. Some categories in VisA, like PCBs, show intricate structures, while others, such as Capsules, consist of multiple objects, making the tasks of anomaly detection and localization quite challenging.

(2) Implementation Details: All input images were resized to 256 × 256 pixels and then center-cropped to 224 × 224 pixels. The adapter module integrated multi-scale features from the backbone, incorporated spatial information via coordinate convolution, and applied pooling operations while maintaining consistency in input–output dimensions. All experiments were conducted on a Windows 11 system equipped with an NVIDIA RTX 4090 GPU (24 GB VRAM), an Intel i7-13700K CPU, and 32 GB of RAM. The model was optimized using the Adam optimizer [40] with a learning rate of 0.0001 and a weight decay of 0.0005. The batch size was set to 32. For the loss functions, the weights were configured as follows: in Stage 1, the attraction loss weight α=1.0, separation loss weight β=0.1, and entropy loss weight γ=0.01; in Stage 2, the attraction loss weight δ=1.5. The margin for the contrastive loss *m* was set to 0.1. The optimal weight for the contrastive loss λ was determined to be 0.8 through an ablation study detailed in Section 3.2.

In terms of computational complexity, AnomNet maintained high efficiency during inference, which is critical for industrial applications. The inference time was dominated by a single forward pass through the network, followed by an efficient nearest-centroid search with a complexity of O(N·K·C), where *N* is the number of feature vectors and *K* is the number of centroids. The training phase was more intensive, particularly in Stage 2, where the contrastive loss computation involved creating a pairwise similarity matrix, resulting in a quadratic complexity of $O(N^{2}\cdot C)$. However, Stage 1 remained efficient, with a complexity linear to the number of features, and the overall training cost was a manageable trade-off for the resulting high performance.

### 3.2. Ablation Study

To evaluate the effectiveness of the proposed method and assess the contribution of each individual component, we conducted a comprehensive ablation study. The study was designed to systematically validate the benefits of our dual-stage strategy and the specific roles of the separation loss ($L_{sep}$), entropy loss ($L_{ent}$), and contrastive loss ($L_{con}$). The results are summarized in Table 1.

The results presented in Table 1 systematically deconstruct the contributions of our framework’s components. First, we analyzed the single-stage strategy, presented as models (a) and (b) in Table 1, which represents joint optimization. The comparison between model (a) and (b) isolated the effect of the contrastive loss in this setting, showing that its inclusion improved the I-AUROC from 97.7% to 98.1%. This demonstrates that contrastive learning is beneficial, though the overall performance of this strategy remains suboptimal. Our analysis then focused on the dual-stage strategy, corresponding to models (c)–(g) in Table 1. The baseline dual-stage model (c), trained only with attraction loss, performed poorly (96.5% I-AUROC) due to severe centroid collapse, highlighting the necessity of regularization in Stage 1. Introducing only the entropy loss (d) or only the separation loss (e) both led to significant performance gains, with the separation loss proving more critical (98.8% vs. 98.1%). This confirms their essential roles in preventing centroid collapse and promoting centroid diversity. Combining both regularization losses (f) further improved performance to 99.3%, establishing the optimal configuration for Stage 1. Finally, applying the Stage 2 contrastive loss to this optimal configuration yielded our full AnomNet model (g), which achieved the best performance (99.5%). This demonstrates the crucial contribution of the contrastive learning stage in refining the feature space after stable centroids have been established.

**Figure 2:**
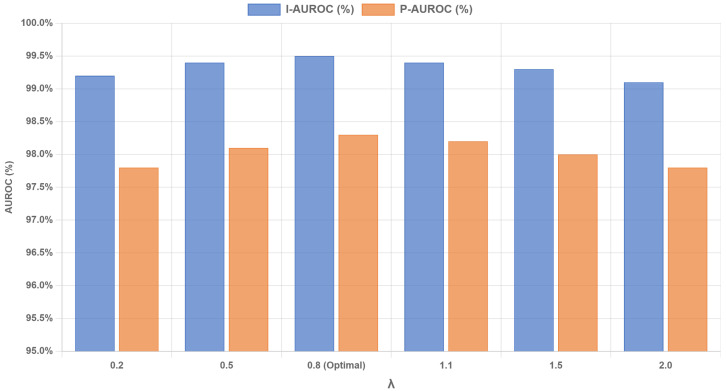
Ablation study on the contrastive loss weight λ. The performance on both image-level and pixel-level AUROC peaks when λ=0.8.

Furthermore, to determine the optimal value for the contrastive loss weight λ and analyze its effect on performance, we conducted an additional ablation study. This parameter is crucial as it balances the feature refinement process in Stage 2. We varied λ and evaluated its impact on performance, with the results presented in Figure 2. The study shows that the model’s performance peaks at λ=0.8. When λ is too small, the contrastive learning effect is insufficient for optimal boundary refinement. Conversely, when λ is too large, the excessive penalty on negative pairs may disrupt the learned feature structure. This experiment confirms that λ=0.8 provides the optimal balance and was used for all reported experiments.

**Figure 3:**
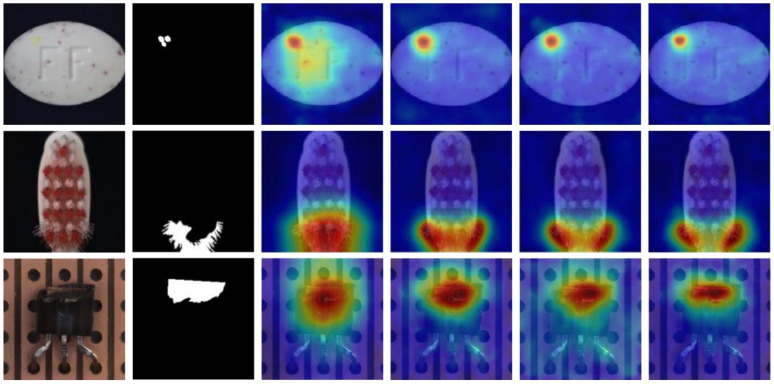
Visual comparison of ablation study results. Each row displays the input image (column 1) and the corresponding ground truth (column 2), followed by the results of four different strategy configurations: the single-stage baseline (column 3), single-stage with contrastive loss (column 4), dual-stage baseline (column 5), and the proposed dual-stage method with contrastive learning (column 6).

Figure 3 presents a comparative visualization between our method and a simplified single-stage variant without contrastive learning. The comparison revealed significant improvements in localization precision and boundary clarity when contrastive learning was incorporated. The single-stage approach produced diffuse and less accurate heatmaps with considerable background noise, while our two-stage contrastive method generated sharp, well-defined anomaly boundaries with minimal false activations. This visual evidence reinforces the effectiveness of our architectural design in enabling accurate and reliable anomaly localization.

### 3.3. Quantitative Results

We evaluated our method using the area under the receiver operating characteristic curve (AUROC) both at the image and pixel level. For anomaly detection, we calculated the area under the receiver operating characteristic curve for detection (I-AUROC) using the produced anomaly detection scores. For anomaly localization, we used the anomaly map to evaluate the pixel-wise area under the receiver operating characteristic curve, denoted as P-AUROC. Given that anomalous pixels typically constitute a small fraction of the image, we further evaluated PRO (Per-Region Overlap) scores to assess the pixel-level localization.

As shown in Table 2, we conducted a detailed comparison of image-level anomaly detection and pixel-level anomaly localization with different methods on the MVTec AD dataset.

For image-level anomaly detection, our method achieved the highest Image AUROC in 7 out of 15 categories, demonstrating exceptional global discriminative capability. It performed particularly well on texture categories such as grid and leather. Compared with SimpleNet, the best-performing embedding-based method, our approach was only marginally lower (0.1%) in average object precision (Avg. Obj) while exhibiting superior performance in several key categories, indicating better robustness while maintaining a high accuracy.

For pixel-level anomaly localization, our method demonstrated outstanding performance among all comparative approaches. Specifically, among embedding-based methods, our Pixel AUROC reached 98.3%, achieving the current SOTA with a 0.2% improvement over SimpleNet. Compared to the current best-performing GLAD (99.3% I-AUROC/98.7% P-AUROC), although our P-AUROC was slightly lower, we achieved a 0.2% improvement in I-AUROC, demonstrating more refined anomaly detection capabilities. Additionally, we further evaluated PRO scores on the MVTec dataset, ultimately achieving an average PRO score of 93.3%.

As shown in Table 3, our method achieved a 97.1% and 98.6% performance for the I-AUROC and P-AUROC metrics, respectively. Although GLAD led in image-level anomaly detection (I-AUROC) with 99.5%, our method tied for the best in pixel-level anomaly detection (P-AUROC) at 98.6%, significantly outperforming other existing methods such as DRAEM, CSFlow, PaDiM, PatchCore, and SimpleNet, demonstrating the effectiveness of our approach in anomaly detection tasks.

In summary, our method exhibits strong generalization ability and superior accuracy in both image-level and pixel-level tasks. Notably, it achieves these results without relying on reconstruction or additional auxiliary networks, outperforming several mainstream approaches. These findings validate the effectiveness of our proposed two-stage training strategy and centroid-driven optimization mechanism in unsupervised anomaly detection settings.

### 3.4. Visualization Results

**Figure 4:**
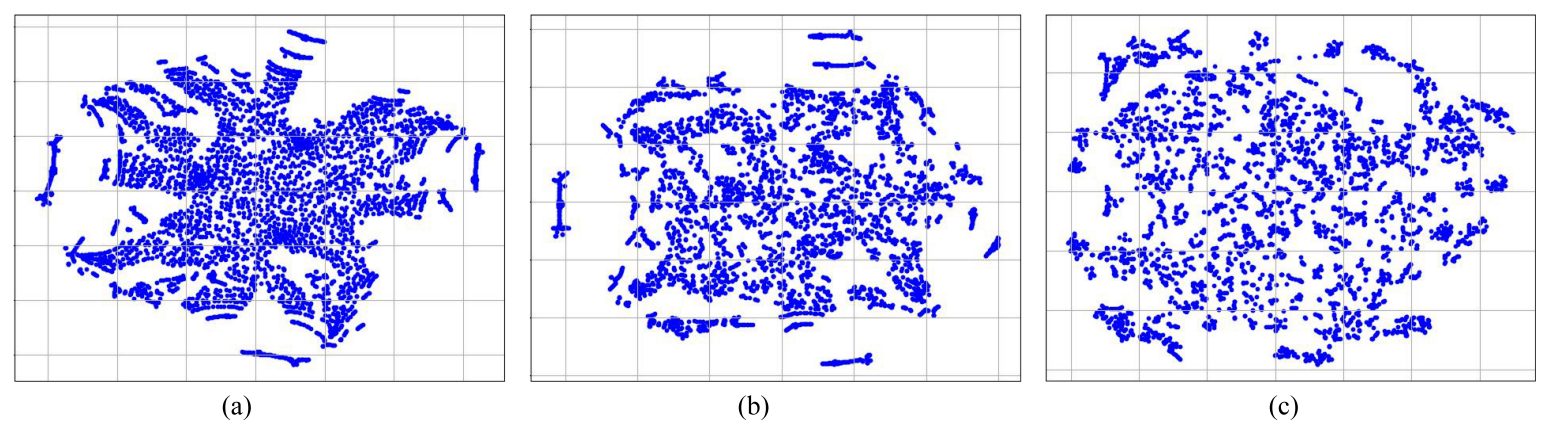
Evolution of centroid distribution during the first stage of centroid optimization. (**a**) Initial centroid distribution with concentrated positions and limited spatial coverage; (**b**) mid-training state showing gradually increasing distances between centroids and preliminary cluster separation; (**c**) final state at the end of the first stage, where centroids are dispersed in an orderly manner.

Figure 4 illustrates the dynamic evolution of multiple centroids within the embedding space during the initial stage of the proposed centroid optimization process. This visualization captures how the model leverages separation loss and entropy regularization in early training to encourage the dispersion of centroids, progressively shaping a more discriminative and structured clustering configuration.

Specifically, Figure 4a depicts the initial state of centroid distribution, characterized by concentrated positions and limited spatial coverage. Figure 4b reflects the mid-training phase, during which the distances between centroids gradually increase, indicating the onset of cluster separation. Finally, Figure 4c presents the outcome at the end of the first stage, where the centroids exhibit a more dispersed and orderly arrangement, signifying an enhanced representational capacity and improved clustering quality.

**Figure 5:**
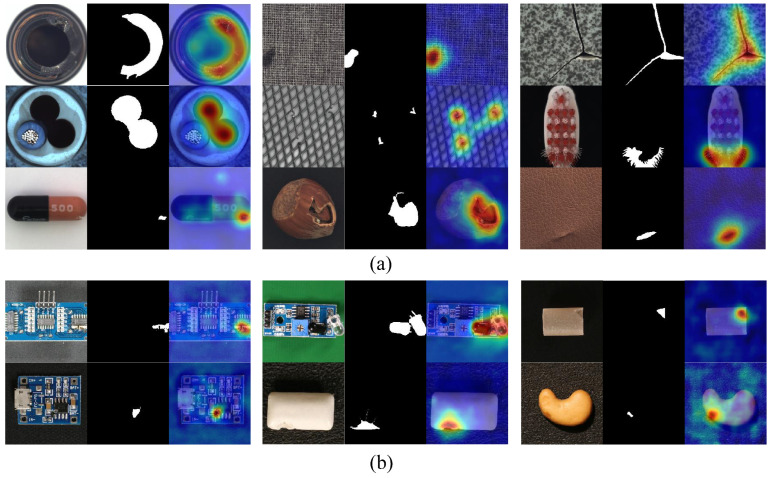
Anomaly localization visualization on MVTec AD (**a**) and VisA (**b**) datasets. Each triplet displays anomaly images (**left**), ground truth (**center**), and predicted heatmap (**right**).

To evaluate the efficacy of our approach in anomaly localization, we selected representative samples from multiple categories for qualitative analysis. Figure 5 presents the anomaly localization visualization results on several categories from the MVTec-AD (a) and VisA (b) datasets. The visualizations comprise three key components: anomalous input images, corresponding ground truth masks, and localization heatmaps generated by our model. The heatmaps are derived from pixel-level anomaly scores, where warmer colors (red/yellow) denote higher anomaly likelihoods and cooler colors (blue) indicate normal regions.

Our observations demonstrate that the proposed method accurately localized anomalous regions across diverse categories, with localization boundaries closely aligned with ground truth anomalies. The heatmaps exhibit distinct high-response regions (red) corresponding to defect areas, while maintaining low responses in background regions, reflecting the model’s strong spatial resolution and anomaly discrimination capabilities. Importantly, the localization results reveal fine-grained detection performance, effectively capturing both large-scale defects (e.g., broken components) and subtle anomalies (e.g., surface scratches and texture irregularities).

More specifically, the method consistently localized anomalies in both texture-based and object-based categories, indicating robust generalization across varied industrial scenarios. For texture categories such as carpet and leather, the model successfully identified disruptions in patterns and surface inconsistencies. For object categories including bottle and metal nut, structural defects such as cracks, holes, and deformations were precisely detected. The high concordance between our model’s localization outputs and the ground truth masks, demonstrated by precise boundary alignment and minimal false positives, further validates the effectiveness of our approach.

Collectively, these visualization results confirm that our method not only achieves reliable anomaly detection but also provides precise and interpretable localization of anomalous regions. This capability offers an explainable solution suitable for industrial inspection applications, where clear and accurate visual feedback facilitates rapid defect identification and supports efficient decision-making in quality assurance processes.

## 4. Conclusions

In this paper, we introduce AnomNet, an unsupervised anomaly detection framework tailored for industrial inspection. Our method combines a lightweight feature adaptation module with a novel dual-stage centroid optimization strategy to address domain shifts and model complex feature distributions. The addition of centroid-based contrastive learning further sharpens feature boundaries and improves anomaly discrimination. Extensive experiments on the challenging MVTec AD and VisA datasets confirmed the robustness and superior performance of AnomNet in both image-level anomaly detection and pixel-level localization. Despite these encouraging results, several limitations remain. The current framework focuses on static images, limiting its effectiveness in capturing process-related anomalies in video streams. Incorporating temporal modeling will be an important step forward. Moreover, although inference is efficient, the quadratic complexity of the contrastive learning stage increases training costs, motivating the search for more scalable solutions. Finally, future work will investigate integrating the framework with federated learning to enable privacy-preserving, multi-site collaboration.

## Figures and Tables

**Table 1:** Comprehensive ablation study of AnomNet components on the MvTec AD dataset.

Model	Strategy	$L_{\mathrm{sep}}$	$L_{\mathrm{ent}}$	$L_{\mathrm{con}}$	I-AUROC (%)	P-AUROC (%)
(a)	Single-stage	✓	✓		97.7	96.9
(b)	Single-stage	✓	✓	✓	98.1	97.2
(c)	Dual-stage				96.5	96.2
(d)	Dual-stage		✓		98.1	97.0
(e)	Dual-stage	✓			98.8	97.5
(f)	Dual-stage	✓	✓		99.3	97.8
(g)	Dual-stage	✓	✓	✓	99.5	98.3

**Table 2:** Comparison with state-of-the-art methods on MVTec-AD. I-AUROC (%)/P-AUROC (%) are reported.

Taxonomy	Reconstruction-Based			Embedding-Based				Ours
Method	DR/EM [6]	RD4AD [7]	GLAD [8]	PaDiM [24]	CSFlow [19]	PatchCore [25]	SimpleNet [26]	Anomnet
Carpet	97.0/95.5	98.8/98.9	99.0/98.5	99.8/99.1	100/-	98.7/99.0	99.7/98.2	99.6/98.7
Grid	99.9/99.7	100/99.3	100/99.6	96.7/97.3	99.0/-	98.2/98.7	99.7/98.8	100/98.5
Leather	100/98.6	100/99.4	100/99.8	100/99.2	100/-	100/99.3	100/99.2	100/99.0
Tile	99.6/99.2	99.3/95.6	100/98.7	98.1/94.1	100/-	99.2/95.0	100/94.5	99.7/94.7
Wood	99.1/96.4	99.2/95.3	99.4/98.4	99.2/94.9	100/-	99.2/95.0	100/94.5	99.7/94.7
Avg. Text	99.1/97.9	99.5/97.7	99.7/99.0	95.5/96.9	99.8/-	99.0/97.5	99.8/97.5	99.7/97.3
Bottle	99.2/99.1	100/98.7	100/98.9	99.1/98.3	99.8/-	100/98.6	100/98.0	100/98.6
Cable	91.8/94.7	95.0/97.4	99.9/98.1	97.1/96.7	99.1/-	99.5/98.4	99.9/97.6	99.9/98.8
Capsule	98.5/94.3	96.3/98.7	99.5/98.5	87.5/98.5	97.1/-	98.1/98.8	97.7/98.9	98.1/98.8
Hazelnut	100/99.7	99.9/98.9	100/99.5	99.4/98.2	99.6/-	100/98.7	100/97.9	100/98.5
Metal Nut	98.7/99.5	100/97.3	100/98.8	96.2/97.2	99.1/-	100/98.4	100/98.8	100/99.0
Pill	98.9/97.6	99.6/98.2	98.1/97.9	90.1/95.7	98.6/-	96.6/97.4	99.0/98.6	98.6/98.8
Screw	93.9/97.6	97.0/99.6	96.9/99.1	97.5/98.5	97.6/-	98.1/99.4	98.2/99.3	98.1/98.8
Toothbrush	100/98.1	99.5/99.1	100/99.4	100/98.8	91.9/-	100/98.7	99.7/98.5	100/98.8
Transistor	93.1/90.9	96.7/92.5	98.3/96.2	94.4/97.5	99.3/-	100/96.3	100/97.6	99.8/98.5
Zipper	100/98.8	98.5/98.2	98.5/97.9	98.6/98.5	99.7/-	99.4/98.8	99.9/98.9	99.6/98.7
Avg. Obj	97.4/97.0	98.0/97.9	99.1/98.4	96.0/97.8	98.2/-	99.2/98.4	99.5/98.4	99.4/98.7
Average	98.0/97.3	98.5/97.8	99.3/98.7	95.8/97.5	98.7/-	99.1/98.1	99.6/98.1	99.5/98.3

**Table 3:** Comparison results on the VisA dataset. I-AUROC (%) and P-AUROC (%) are reported.

Method	DR/EM	CSFlow	PaDiM	PatchCore	SimpleNet	GLAD	Ours
I-AUROC (%)	81.8	75.8	78.1	90.3	89.2	99.5	97.1
P-AUROC (%)	78.1	95.6	95.9	96.8	95.3	98.6	98.6

## Data Availability

Publicly available datasets were analyzed in this study. These data can be found at: MVTec AD Dataset: https://www.mvtec.com/company/research/datasets/mvtec-ad (accessed on 21 July 2025) and VisA Dataset: http://github.com/amazon-research/spot-diff (accessed on 21 July 2025).

## Abbreviations

The following abbreviations are used in this manuscript:

DSCO	Dual-Stage Centroid Optimization
CCL	Centroid-based Contrastive Learning
CoordConv	coordinate convolution
AUROC	Area Under the Receiver Operating Characteristic Curve
I-AUROC	Image-level Area Under the Receiver Operating Characteristic Curve

p-AUROC	Pixel-level Area Under the Receiver Operating Characteristic Curve
PRO	Per-Region Overlap
